# How Does the Level of Physical Activity Influence Eating Behavior? A Self-Determination Theory Approach

**DOI:** 10.3390/life13020298

**Published:** 2023-01-20

**Authors:** Vanessa Fernandes, Filipe Rodrigues, Miguel Jacinto, Diogo Teixeira, Luís Cid, Raul Antunes, Rui Matos, Rafael Reigal, Antonio Hernández-Mendo, Verónica Morales-Sánchez, Diogo Monteiro

**Affiliations:** 1 ESECS—Polytechnic of Leiria, 2411-901 Leiria, Portugal; 2 Life Quality Research Centre (CIEQV), 2040-413 Leiria, Portugal; 3 Faculty of Physical Education and Sport, Lusófona University (FEFD-ULHT), 1749-024 Lisbon, Portugal; 4 Research Center in Sport, Physical Education, Physical Activity and Health (CIDEFES), 1749-024 Lisbon, Portugal; 5 Sports Science School of Rio Maior—Polytechnic Institute of Santarém, 2040-413 Rio Maior, Portugal; 6 Research Center in Sport Sciences, Health Sciences, and Human Development (CIDESD), 5001-801 Vila Real, Portugal; 7 Center for Innovative Care and Health Technology (ciTechCare), Polytechnic of Leiria, 2411-901 Leiria, Portugal; 8 Department of Social Psychology, Social Work, Social Anthropology and East Asian Studies, University of Málaga, 29016 Málaga, Spain

**Keywords:** physical activity, eating behavior, regulation of eating behavior

## Abstract

Physical activity and diet are two predominant determinants of population health status that may influence each other. Physical activity has been identified as a behavior that may lead to a healthier diet and regulates eating behaviors. This research aimed to investigate how the level of physical activity is associated with the motivation related to eating behaviors and, consequently, the eating style individuals have on a daily basis. This was a cross-sectional study in which participants completed an online questionnaire that assessed the following variables: the level of physical activity, the motivation toward eating behavior, and the type of eating behavior. In total, 440 individuals (180 men and 260 women) who regularly exercised in gyms and fitness centers aged between 19 and 64 years (M = 33.84; SD = 10.09) took part in the study. The data were collected following the Declaration of Helsinki and with the approval of the Ethics Committee of the Polytechnic of Leiria. For the statistical analysis, mean and standard deviations were first calculated, as well as bivariate correlations between all the variables of interest. Then, structural equation model analyses were performed considering the levels of physical activity as the independent variable, motivations toward eating behavior as the mediators, and eating styles as the dependent variables. It was concluded that a greater level of physical activity leads to a more self-determined type of eating regulation, which in turn results in less constricted eating behaviors that are influenced by external factors and emotional factors.

## 1. Introduction

Low levels of physical activity and dietary habits that deviate from the recommendations are major contributors to the prevalence of chronic diseases that widely negatively influence the global population in terms of health [1,2]. Individuals who eat healthier diets and adhere to physical activity recommendations have more favorable health outcomes such as longevity, mental health, and a lower risk of chronic diseases such as diabetes type II, hypertension, and obesity [3,4]. Physical activity is recognized as a factor that leads to lower morbidity and mortality from various causes, as well as weight control [5]. Physical activity improves several health indicators such as blood pressure, resting heart rate, waist circumference, visceral fat, insulin sensitivity, leptin sensitivity, blood lipid levels, physical fitness, body composition, and also psychological health indicators such as an improved mood [5,6]. In addition, due to physiological aging, muscle and strength loss (i.e., sarcopenia) occur due to a lack of physical stimuli. Thus, physical activity can maintain or even improve physical fitness in aging adults [7].

Maintaining healthy eating habits is not an easy task nowadays due to the obesogenic environment in which individuals have easy access to high-caloric and highly palatable foods. That combined with low levels of physical activity leads to a greater risk of previously mentioned health problems. The increasing tendency of the population to be overweight and obese indicates that the energy intake chronically exceeds the daily energy expenditure. Although the benefits of a diet that promotes a healthy weight are obvious, many people do not adhere to these recommendations, and dietary interventions alone may not be sufficient to change eating behaviors [2,6,8]. Applying theories of behavior change, such as the theory of self-determination, can lead to the long-term maintenance of this change, since theoretical behavior change models can provide evidence on how to create efficient strategies on promoting healthy behaviors such as balanced dieting [9].

Self-determination theory, proposed by Deci and Ryan [10], states that humans have a natural inclination to act in line with their motivational state in a given setting. This theoretical paradigm focuses on contextual circumstances and the causes and consequences of self-determined behavior. This framework distinguished self-determined motivation from non-self-determined motivation based on the quality of motivation rather than quantity [10]. Self-determined motivation assumes that the behavior is performed due to the positive values inherent in the behavior, while the person integrates the behavior into his/her daily lives, and includes identified regulation, integrated regulation, and intrinsic motivation, the three most self-determined forms of motivation present on the motivational continuum. This motivation is described as participating in a conduct that is viewed as being congruent with intrinsic goals or outcomes and stems from the individual’s identity, such as healthy eating. Individuals that engage in self-determined motivational actions have a sense of choice, interest, and satisfaction and, as a result, tend to persist in the healthy eating behavior. Non-self-determined motivation assumes that the performance of the behavior is adjacent to coercive or self-imposed assumptions and it includes introjected regulation, external regulation, and in some instances, amotivation, the three least self-determined forms found on the motivational continuum based on the assumptions of the self-determination theory. Individuals who participate in a controlled behavior feel a sense of obligation and pressure and tend to stick with the conduct only as long as the external or self-imposed contingency is present. The action is likely to be abandoned if the stimulus is removed. Individuals who operate on the basis of a regulated incentive are thus less likely to be self-determined in the long run [10]. The response of individuals exposed to an adipogenic environment varies because not all individuals become obese when exposed to it. Some individuals may be genetically predisposed to become obese because they possess the fat mass and obesity-associated protein (i.e., FTO gene), which increases appetite. However, in more active individuals, the effect of this gene has been reversed, leading to the assumption that physical activity can offset the biologically determined propensity to obesity [6]. In a recent study, physical activity was positively associated with a greater correspondence between food need and food intake; that is, a greater perceived control in following the sensation of hunger and satiety. These results confirm previous studies which suggested physical activity as an entry point to a healthier diet [11]. Approaches that intuitively and cognitively stimulate the homeostatic regulation of food intake through hunger and satiety signals have been explored and have gained recognition. Intuitive eating is associated with fewer eating disorders and greater well-being [12].

Physical activity has been highlighted as behavior that leads to a healthier diet and is essential in regulating weight in various populations, including individuals of a normal weight. This behavior influences physiological processes such as appetite control and psychological aspects such as self-efficacy and body image, leading to greater self-determined motivation and, consequently, improvements in dietary self-regulation [3,11]. Higher levels of physical activity may have greater effects on eating behavior motivation. Physical activity may lead to a higher rate of weight loss success, in part because of its effects on eating behavior, such as more flexible food constraints and a lower pattern of emotional eating [13]. On the other hand, a lower involvement in physical activity is associated with extrinsically motivated eating behavior and is associated with a higher body mass index (BMI) as described by Castro et al. [14].

### Current Research

Further studies are needed to explore the mechanisms involved in appetite control that account for differences between individuals, such as body composition, postprandial satiety, hunger signaling peptides, insulin and possibly leptin sensitivity, gastric emptying, and the basal metabolic rate [15]. The possible role of appetite-controlling peptides such as CCK, GLP-1, PYY, ghrelin, or recently discovered nesfatin-1 (see Prinz et al. [16]) could have its basis on physical active behaviors due to the implications of physiological changes due to exercise. The effect of exercises on the levels of appetite-related hormones and appetite have also been described [17], suggesting that physically active behaviors can lead to motivations that are in line with healthy habits since physiological changes occur at the same degree [18]. The differences in body composition and insulin sensitivity may be factors that promote a more sensitive appetite control in physically active individuals [5].

It is known that as people become more physically active, they move from an unregulated to a regulated zone of appetite control and better match their energy expenditure to their needs [1,6]. However, the levels of physical activity that can induce the greatest physiological and behavioral changes concerning motivation and eating behaviors remains understudied [14]. Physical activity may be a major factor in preventing overweight and obesity as well as in the regulation of eating behavior even in people with healthy weights, according to the evidence [11,14]. Physical activity may be a motivational driver of a healthier eating style and also help to regulate food so that it suits individual needs. Distinct degrees of physical activity might also have different effects on how people regulate their eating behavior. This research aimed to investigate how the level of physical activity is associated with the motivation related to eating behaviors and, consequently, the eating style individuals have on a daily basis. In light of the existing literature, a hypothesis was created. Greater levels of physical activity would be positively and significantly associated with self-determined eating behavior and negatively associated with non-self-determined eating behavior. The second hypothesis is that self-determined eating behavior would be negatively associated with external ingestion, emotional ingestion, and constraint. Last, non-self-determined eating behavior would be positively and significantly related with external ingestion, emotional ingestion, and constraint.

## 2. Materials and Methods

### 2.1. Participants

The minimum sample size needed for this investigation was determined using the a priori sample size calculator for structural equation analysis created by Soper [19]. The inputs for calculating the sample size were determined according to previous research [15], namely the predicted effect size was 0.2, the intended statistical power was 0.95, the probability level was 0.05, there were 5 latent variables, and there were 40 observable variables. The calculator assessed a minimum of 376 participants to provide sufficient statistical power for results to be valid and reliable. In the present study, 440 individuals (180 men and 260 women) who regularly exercise in gyms and fitness centers and were between the ages of 19 and 64 years (M = 33.84; SD = 10.09) took part in the study. They were mostly engaged in resistance training and similar activities such as cross training and weightlifting. Their height varied between 147 and 191 cm (M = 169; SD = 0.09) while their weight ranged between 42 and 127 kg (M = 70.29; SD = 14.37).

### 2.2. Instruments

The International Physical Activity Questionnaire Portuguese Short version [20] was used to assess the level of physical activity. This questionnaire contains eight questions about physical activity performed in the seven days preceding the application of the questionnaire, namely two about vigorous-intensity physical activity, two about moderate-intensity physical activity, two about light-intensity physical activity, and two about sitting time. The total sitting time was not considered in this study. The short version allows for a calculation of the Metabolic Equivalents of Task (MET) which measures the energy expenditure and amount of physical activity. By calculating the duration (in minutes) and frequency (in days) of the three types of physical activity (i.e., total amount of MET-minutes/week = sum of light + moderate + vigorous-intensity scores MET-minutes/week), a total score for the MET was calculated [21].

The Regulation of Eating Behavior Scale Portuguese version [22] was used to assess the eating behavior motivational regulation [23]. This instrument consists of 24 items divided into six factors (four items each), to which individuals respond to each item on a seven-level Likert scale ranging from 1 (“strongly disagree”) to 7 (“strongly agree”). This instrument measures amotivation (e.g., “I honestly don’t know what I get out of this”); external regulation (e.g., “Because people close to me insist that I do it”); introjected regulation (e.g., “Because I feel that I have to be thin”); identified regulation (e.g., “Because it is a way to obtain long-term health benefits”); integrated regulation (e.g., “Because eating healthy is a fundamental part of my life”); and intrinsic motivation (e.g., “Because I enjoy preparing healthy meals”). The subscales showed an adequate internal consistency, specifically amotivation (α = 0.95); external regulation (α = 0.91); introjected regulation (α = 0.88); identified regulation (α = 0.88); integrated regulation (α = 0.91); and intrinsic motivation (α = 0.89). For the purpose of this study, the composite scores for self-determined motivation (i.e., intrinsic motivation, integrated, and identified regulation) and non-self-determined motivation (i.e., introjected regulation, external regulation, and amotivation) were calculated based on previous assumptions [11]. The measurement model of the measure in this study displayed an acceptable fit to the data: χ^2^/df = 1.21, Comparative Fit Index = 0.939, Tucker–Lewis Index = 0.922, Standard Mean Root Square Residual = 0.043, and Root Mean Square Error of Approximation = 0.057 (90% Confidence Interval = [0.050, 0.065]).

The Dutch Eating Behavior Questionnaire Portuguese version [24] was used to assess the types of eating behaviors [25]. This questionnaire contains 33 items to which individuals respond to each item on a Likert-type scale ranging from 1 (“Never”) to 5 (“Very frequently”). Items are grouped into the type of eating behavior, namely: constraint (attempts to avoid food intake—10 items), emotional ingestion (overeating in response to emotions—13 items), and external ingestion (eating in response to food-related stimuli). The factors demonstrated an adequate internal consistency since the scores were above the cutoff, namely constraint (α =0.85), emotional ingestion (α =0.90), and external ingestion (α =0.95). The measurement model of this measure in this study displayed an acceptable fit to the data: χ^2^/df = 1.92, Comparative Fit Index = 0.942, Tucker–Lewis Index = 0.929, Standard Mean Root Square Residual = 0.051, and Root Mean Square Error of Approximation = 0.053 (90% Confidence Interval = [0.043, 0.059]).

### 2.3. Procedures

The data were collected following the Helsinki Declaration [26] and the Ethic Committee of the Polytechnic of Leiria approved its implementation (CE/IPLEIRIA/26/2021). The current study design was cross-sectional and several gym and fitness center club managers in Portugal were contacted to explain the objectives of the study and to request their approval for data collection purposes. After approval, club managers disseminated the questionnaire using their data records. Potential participants were informed about the research objective, the estimated time to complete the questionnaires (approximately 10 min), and all the ethical procedures that were followed and respected. Before completing the questionnaires, participants had to check a box indicating that they understood the objective of the present study and agreed to participate voluntarily in this study. Participants also signed their informed consent. Participants were thanked for their participation, but no compensation was given.

### 2.4. Statistical Analysis

Descriptive statistics such as the means and standard-deviations were calculated as well as the bivariate correlations between all the variables under analysis using the IBM SPSS STATISTICS version 25.0 software (IBM Corp., Armonk, NY, USA). In order to determine the statistical significance of a deviation from normal distribution, the skewness and kurtosis estimates were divided by their corresponding standard error to obtain the z score. A z score below |1.96| suggested a normal distribution. Next, a two-step maximum likelihood approach (ML) was performed using the IBM SPSS AMOS version 23.0 software [24]. First, the measurement model was estimated using confirmatory factor analysis to assess the psychometric properties of the measurement model. The convergent validity was calculated considering the average variance extracted and coefficients ≥ 0.50 were considered as acceptable [27,28]. The discriminant validity was assumed to be adequate when the square of the correlations between the factors was less than the average extracted variance value of each of the factors [27]. Furthermore, the internal consistency of each of the latent variables was calculated using the composite reliability calculator [29], with a coefficient score ≥ 0.70 deemed as acceptable [27,29]. The structural model was then performed to test the proposed associations.

The measurement model and structural model’s suitability were assessed using traditional incremental indices such as the Comparative Fit Index (CFI) and Tucker–Lewis Index (TLI), as well as absolute indices such as the Standardized Root Mean Residual (SRMR) and Root Mean Square Error of Approximation (RMSEA) and its respective confidence intervals, as proposed by several authors [27,30,31,32]. For these indices, the following cutoff values were considered: CFI and TLI ≥ 0.90 and RMSEA and SRMR ≤ 0.08.

The standardized direct and indirect effects on the dependent variables were also investigated. The significance of direct and indirect effects was determined using a bootstrap resampling procedure (1000 bootstrap samples) and a 95% confidence interval (CI). The direct and indirect effects were considered significant if the 95% CI did not include zero [33]. We chose confidence intervals over the probability of significance (*p*-value) due to recent evidence of a mediation without a significant relationship between the variables [34].

## 3. Results

Full information robust maximum likelihood was used to deal with the small amount of missing data at the item level (random missing = 3%), as proposed by Enders [35]. The Mardia coefficient (2333.46) was higher than expected for multivariate normality. As a result, the Bollen–Stine bootstrap was employed in the following analyses [36]. In addition, the variance inflation factors were examined to confirm the possibility of multicollinearity between the variables. The values between the independent variables and dependent variables were all equal to or lower than 2.00, indicating that the multivariate regression model could be run under acceptable conditions [27,30].

The descriptive statistics, bivariate correlations, and internal consistency coefficients are shown in Table 1. The skewness and kurtosis values ranged between −2 and +2, revealing no deviations from univariate normality. Participants reported weekly physical activity levels greater than 1000 MET. Self-determined motivation displayed lower scores (M = compared to non-self-determined motivation). Nonetheless, low levels of external ingestion (M = 2.03; SD = 1.07), emotional ingestion (M = 2.25; SD =0.97), and constraint (M = 2.24; SD = 1.12) were reported. The pattern of correlations displayed significant associations as theoretically expected, namely: (a) the total physical activity was positively associated with self-determined motivation; (b) self-determined motivation was negatively associated with external, emotional, and constraint eating behaviors; and (c) non-self-determined motivation was positively correlated with external, emotional, and constraint eating behaviors.

The measurement model showed that the data fit the model: [χ^2^/df = 4.74 (215), B-S p = 0.001, TLI = 0.918, CFI = 0.929, SRMR = 0.063, RMSEA = 0.068 (IC = 0.056, 0.068)]. The results from the factor loadings revealed adjusted values of convergent validity since the scores for the average variance extracted were above the cutoff (see Table 1), as well as adequate values of the discriminant validity, since the square of the correlations between the factors was less than the value of the average variance extracted from each of the factors as the latent factors also displayed adjusted internal consistency values (>0.70).

**Figure 1:**
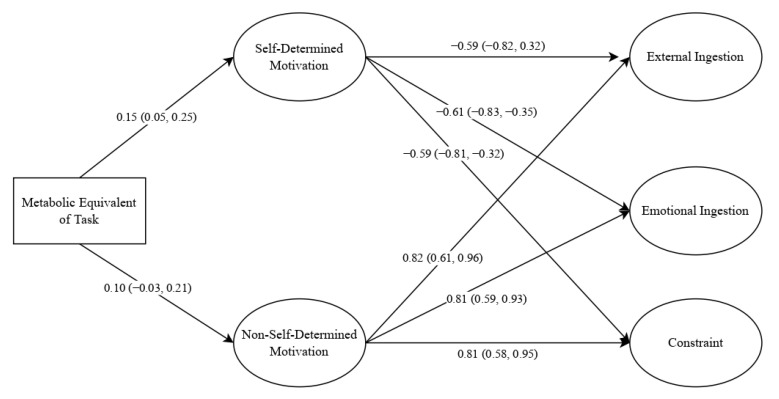
Structural equation model. Notes: standardized coefficients are reported; within brackets = 95% confidence interval.

Regarding structural equation modelling, the proposed model fit the data well: [χ^2^/df = 9.72 (220), B-S p = 0.001, TLI = 0.908, CFI = 0.917, SRMR = 0.068, RMSEA = 0.066 (IC = 0.054, 0.066)]. Figure 1 shows standardized direct effects. The total physical activity was more associated with self-determined motivation compared to non-self-determined motivation. Consequently, self-determined motivation was negatively associated with external ingestion, emotional ingestion, and constraint. In contrast, non-self-determined motivation was associated with external ingestion, emotional ingestion, and constraint. The standardized indirect effects between the total physical activity and external, emotional, and constraint eating behaviors were not significant (see Table 2).

## 4. Discussion

This research aimed to investigate how the level of physical activity is associated with the motivation related to eating behaviors and, consequently, the eating style individuals have on a daily basis. A higher level of physical activity was hypothesized to be positively associated with self-determined eating behavior, and this hypothesis was supported. On the other hand, it was theoretically proposed that greater levels of physical activity should be negatively associated with non-self-determined eating behavior, which as was in part corroborated by the current results. Self-determined motivation was negatively associated with external ingestion, emotional ingestion, and constraint. In contrast, non-self-determined motivation was associated with external ingestion, emotional ingestion, and constraint. The results will be discussed in light of the existing literature.

### 4.1. Associations between Physical Activity and Eating Behavior Motivation

Individuals who engage in more physical activity have a more self-determined regulation of their eating habits, whereas those who engage in less physical activity have a less self-determined regulation of their eating habits. Previous studies have concluded that physical activity is associated with a greater agreement between the nutritional needs and nutritional intake performed, increasing the individual’s confidence in the feeling of hunger and satiety [11]. Carraça et al. [11] concluded in their study that a higher level of physical activity had a positive relationship with self-determined eating behavior. Individuals who are physically active have a greater control over their eating behavior since motivation has its influence on identifying and integrating eating habits that are healthy. This study supported the existing research in normal-weight exercisers and extended it by investigating the motivational regulation, through self-determined motivations, that links physical activity to eating behaviors. It is critical that these findings be duplicated in other populations.

### 4.2. Associations between Eating Behavior Motivation and Types of Eating Behavior

The self-determined regulation of eating habits leads to better types of eating behavior, since the association between self-determined motivation and external ingestion, emotional ingestion, and constraint was significantly negative. Thus, eating that is less influenced by external, emotional factors and has less restrictive attitudes can have a positive impact on the control of binge eating and other compulsive eating disorder. Individuals with non-self-determined regulation for eating habits have greater eating habits that are harmful, that is, external (e.g., eating behavior controlled by significant others) and internal (e.g., perception of a poor body image) contingencies influences more emotional and restrictive eating behavior. Van Strien and Koenders [37] also concluded in their work that physical activity was negatively associated with emotional and external eating, which is similar to the findings of the current study. As a result, physical activity can be viewed as a pathway to a self-determined healthier diet since autonomous motivation leads to behaviors that are consistent with healthy habits [11,13,37].

### 4.3. Practical Implications

Sim et al. [38] observed that sedentary adults appear to change their eating behavior based on their perception of other behaviors, such as physical activity. That is, an increase in exercise practice may lead a person to believe that they do not need to be as careful with food since one habit compensates for the other [38]. This is described by the Compensatory Model of Beliefs in Health [39], which claims that the negative impacts of unhealthy conduct can be compensated by beneficial behavior [39,40]. In some ways, these findings are consistent with the findings of this study as a greater level of physical activity may lead to an individual having less restrictive eating behaviors, but this is not necessarily synonymous with having a less balanced diet as a result of a higher level of physical activity.

Martinez-Avila et al. [40] reported that people who engaged in physical activities increased their emotional eating behavior, which shows that exercise may have negatively influenced the ability to resist emotional cues or eat in reaction to various negative feelings. However, this study employed an intervention study in which subjects had scheduled exercise sessions over 6 months [40] and thus the participants were controlled for their eating behaviors. It is crucial to note that some various elements and processes influence eating behavior and food choice [41], with physical activity being just one of the influential aspects to consider in these interactions, with others perhaps overlapping.

According to the findings of this study, a lack of physical activity can lead to a more restrictive diet, which is connected with overeating and weight gain. Food constraint is frequently associated with an overeating propensity (i.e., food disinhibition), emotional eating, and external eating. Following a weight reduction procedure, the risk of regaining weight can be minimized by increasing physical activity, which not only increases energy expenditure but also reduces feelings of tension and improves emotional well-being [42]. Emotional eating is a type of eating that is influenced by emotions, with or without the stimulation of hunger, and can result in food disinhibition. According to Koenders and Van Strien [43], the favorable relationship between emotional eating and a weight increase was weaker among employees who exercised regularly [43]. Individuals who eat emotionally use food to control their emotions, and physical activity has been identified as a protective factor for this behavior [44,45,46,47]. Long-term patterns of overeating in response to negative emotions or owing to competing goals between dieting and the pleasure of eating can also explain diet failure, regardless of the self-control maintained during dieting. Physical exercise, which is related to improved eating habits, may be a factor that aids a successful long-term weight loss diet [48].

It is well known that restrictive, emotional, and external eating behavior can predispose to a higher caloric intake, particularly from fat- and sugar-rich foods such as sweets, savory snacks, and fast food [49]. Oh and Taylor [50] also discovered that regular chocolate consumers can reduce their cravings with exercise. Increasing physical activity by encouraging better eating habits (i.e., less restrictive), emotional and external, can lead to better food choices. Food intake is the result of complex interactions between several factors, and evidence of a link between eating behavior and food intake is still lacking [49,50]. As a result, different studies reach different conclusions. Martinez-Avila et al. [40] concluded that healthy young adults with a higher level of physical activity report a tendency to eat compulsively and uncontrollably when developing a study that aimed to associate eating behavior with the time of a sedentary lifestyle and physical activity. According to the authors, this behavior can be explained by the fact that individuals perceive higher levels of physical activity as a reward. However, before concluding that exercise caused this compulsive food consumption, it will be necessary to consider whether individuals are eating according to their needs. This result may be motivated by individuals not having a diet tailored to their nutritional needs, which may result in an increased food intake response at certain times.

### 4.4. Limitations and Agenda for Future Research

The fact that the data were self-reported and collected online is a clear limitation, but this data collection approach allowed for collecting data from a large sample. In addition, due to the sample’s characteristics, we were unable to explore possible differences across age groups and sex. Thus, we suggest that future studies explore possible moderation variables (e.g., sex, age, gym facility, and exercise activity) that could influence the current results. The International Physical Activity Questionnaire Short version is a validated and simple method for estimating physical activity levels. However, it is not an objective assessment and only considers a limited period (only 7 days). Furthermore, only the MET was assessed, with no consideration given to other characteristics such as sedentary behaviors. This study was cross-sectional, which is also a limitation due to the fact that individuals were evaluated in a single moment, which may not fully reflect their eating behaviors. It is worth emphasizing the scarcity of longitudinal and experimental research that investigates how interventions grounded in self-determination theory could enhance physical activity as a mean to increase healthy eating habits.

## 5. Conclusions

A greater dose of physical activity is positively and significantly associated with self-determined eating behavior. In turn, greater self-determined eating behavior is negatively associated with external ingestion, emotional ingestion, and constraint types of eating behavior. Thus, less restrictive eating behavior is less associated with environmental and emotional factors. This study demonstrates that a greater level of physical activity is a crucial element in making it easier to have more self-determined eating habits and favorable eating behavior, which may be a strategy to lower the risk of problems such as obesity and eating disorders, as stated in the literature.

## Figures and Tables

**Table 1:** Descriptive statistics, bivariate correlations, average variance extracted, and composite reliability coefficients.

Variables	M	SD	1	2	3	4	5	6	AVE	CR
1. MET	3269.76	2834.24	1	-	-	-	-	-	-	-
2. Self-determined motivation	4.25	0.64	0.16 **	1	-	-	-	-	0.61	0.82
3. Non-self-determined motivation	4.49	0.82	0.10 *	0.72 **	1	-	-	-	0.58	0.80
4. External ingestion	2.03	1.07	−0.03	−0.69 **	0.64 **	1	-	-	0.85	0.96
5. Emotional ingestion	2.25	0.97	−0.05	−0.71 **	0.67 **	0.88 **	1	-	0.90	0.98
6. Constraint	2.24	1.12	−0.02	−0.73 **	0.70 **	0.91 **	0.87 **	1	0.77	0.93

Notes: M = mean; SD = standard deviation; AVE = average variance extracted; CR = composite reliability coefficients; * *p* < 0.05; ** *p* < 0.01.

**Table 2:** Indirect effects of the variables under study.

Regression Path	Indirect			
	β	IC95%	p	
Metabolic equivalent of task → constraint	−0.009	−0.072, 0.050	0.787	
Metabolic equivalent of task → emotional ingestion	−0.012	−0.076, 0.047	0.729	
Metabolic equivalent of task → Constraint	−0.008	−0.073, 0.052	0.001	

Notes: β = standardized coefficient; 95%CI = 95% confidence interval; p = significance level.

## Data Availability

The data were used under license exclusively for the current study. The data that support the findings of this study are not publicly available but are available upon reasonable request and with permission of the Life Quality Research Center and the corresponding author.
